# Effects of fancy rope-skipping on motor coordination and selective attention in children aged 7–9 years: a quasi-experimental study

**DOI:** 10.3389/fpsyg.2024.1383397

**Published:** 2024-08-07

**Authors:** Libo Deng, Hua Wu, Hui Ruan, Dan Xu, Shibo Pang, Min Shi

**Affiliations:** ^1^Faculty of Physical Education, Hainan Normal University, Haikou, China; ^2^Faculty of Mathematics and Statistics, Yulin Normal University, Yulin, China; ^3^Faculty of Sports Training and Education, Hainan Provincial Sports Academy, Haikou, China; ^4^Faculty of Physical Education and Health, Hainan College of Economics and Business, Haikou, China; ^5^School of Public Education, Hainan College of Software Technology, Qionghai, China

**Keywords:** jump rope, primary school children, cognitive skills, motor skills, d2 Test of Attention, Körperkoordinations Test für Kinder

## Abstract

**Introduction:**

Recent studies have emphasized the intricate connection between exercise and cognition, focusing on specific cognitive processes and their correlations with specific motor skills. However, research on the impact of the qualitative aspects of movement on both short- and long-term cognitive performance is limited. In this quasi-experimental study, we investigate the impact of a 10-week fancy rope-skipping intervention on motor coordination and selective attention of 7–9-year-old children.

**Methods:**

A total of 60 primary school students from Changbin School in Haikou participated and completed the study from October to December 2022. The 60 participants were divided into a fancy rope-skipping group and a control group. Children's motor coordination was assessed using the Körperkoordinations Test für Kinder (KTK), while selective attention was evaluated using the d2 Test of Attention. Children were assessed at baseline and after the 10-week intervention.

**Results:**

Compared with the control group, the scores for the total KTK and for the hopping for height, jumping sideways, and moving sideways sub-items were significantly higher in the rope-skipping group after the intervention, with a significant interaction effect between time and intervention. Attention concentration improved in the rope-skipping group and had a significant interaction effect between time and intervention compared with the control group; the effects of the intervention on other aspects of selective attention were unclear.

**Conclusions:**

Our study suggests that a 10-week fancy rope-skipping intervention may potentially enhance motor coordination and selective attention accuracy in children aged 7–9 years.

## 1 Introduction

Fostering cognitive skills is crucial for children's academic success and overall developmental wellbeing. Selective attention is of particular importance, as a cognitive mechanism that adeptly filters pertinent information while inhibiting interference. Isbell et al. (2017) considered selective attention as a “multiplier” that can significantly influence various cognitive domains, and it is a foundational capacity connected to a range of cognitive abilities and academic success (Páez-Maldonado et al., 2020). Deficits in selective attention have been associated with learning difficulties and behavioral problems in the classroom (Mueller et al., 2017). Research suggested that selective attention stabilizes around the age of 7 and continues to develop throughout ages 8 or 9 (Plebanek and Sloutsky, 2019; Phelps et al., 2022). Training can enhance selective attention (Itthipuripat et al., 2017), highlighting the importance of exploring interventions to improve it in children.

Previous studies have suggested that exercise has a close association with various aspects of cognition (Biddle et al., 2019). A recent systematic review proposes that physical activity is beneficial for cognitive improvement in children and adolescents, and that extracurricular physical education interventions and programs that increase physical activity are the most effective (Álvarez-Bueno et al., 2017). Motor and cognitive skills may share a similar neural basis, with co-activation between the prefrontal cortex, cerebellum, and basal ganglia during some motor and cognitive tasks (Middleton and Strick, 2000). Further, motor and cognitive skills share cognitive processes. Exercise can enhance brain functioning and promote neural plasticity, which may lead to improvements in attentional control (Best, 2010). Additionally, exercise has been found to increase the levels of neurotransmitters, such as dopamine, which are associated with attention and executive functioning (Chang et al., 2012). Children may also experience parallel developmental stages in motor and cognitive skills, with overlapping developmental schedules that accelerate between the ages of 5 and 10 years (van der Fels et al., 2015). However, given the broad domains encompassing both motor skills and cognitive abilities, there is no consensus on this correlation, and recent studies have highlighted an intricate relationship between specific cognitive processes and particular types of motor skills.

Some studies find that the motor dimensions most closely related to cognitive performance are motor coordination (Vojtíková et al., 2023). Motor coordination is a complex collaborative process involving the nervous and musculoskeletal systems. Its core component reflects the degree of body control, which is the basis for learning and mastering motor skills (Vandorpe et al., 2012). van der Fels et al. (2015) proposed that complex motor skills such as fine motor skills, coordinated movements, and sequential movements should be incorporated into motor intervention programs to stimulate motor and higher-order cognitive skills in prepubertal children. Moreover, complex sports, such as ball games, pose more challenges to motor coordination and cognitive processes (Moratal et al., 2020). Howard (2023) also suggests that the focus of attention significantly influences motor coordination.

Fancy rope-skipping is also a physical activity that involves performing a variety of complex and rhythmic jump rope movements that may demand high levels of motor coordination and cognitive processing (Lorke et al., 2022; Burdack and Schöllhorn, 2023). Many studies found that jumping rope has a positive impact on children and adolescents' physical fitness, such as speed, flexibility, strength, coordination (Barrio et al., 2023; Zhao et al., 2023). While research has explored various physical activities' effects on children's development, limited studies have specifically investigated the impact of jumping rope on motor coordination and attention in children aged 7–9 years. We hypothesize that a 10-week intervention involving fancy rope-skipping could significantly enhance both motor coordination and selective attention abilities in this age group. To test this hypothesis, our study aims to assess the effects of the intervention on motor coordination and selective attention performance in school-aged children using the Körperkoordinations Test für Kinder (KTK) and the d2 Test of Attention, respectively.

## 2 Methods

### 2.1 Participants

Following a previous study (Delin et al., 2019), WinPepi software was used to calculate the sample size for a 5% significance level, 80% statistical power, 95% confidence level, and 10% dropout rate; the minimum required sample size was determined to be 50 individuals. Given the potential risk of withdrawal during the pandemic, a total of 60 individuals were recruited as study participants, with 30 allocated to the fancy rope-skipping group and 30 to the control group. These participants were primary school students aged 7–9 years at Changbin Primary School in Haikou City, Hainan Province, China.

The fancy rope-skipping group was selected from students who enrolled in the fancy rope-skipping course after-school program, with inclusion criteria including: (1) healthy children aged 7–9 years without significant illnesses or disabilities, who could participate in fancy rope-skipping activities; (2) those who had not previously received fancy rope-skipping training. The inclusion criteria for the control group were as follows: (1) healthy children aged 7–9 without attend the fancy rope-skipping training; and (2) able to consistently attend physical education. Exclusion criteria included: (1) students participating in other after-school physical education training courses (2) students participating in courses related to improving motor coordination and selective attention; and (3) children with attention deficit disorders, neurological and developmental disorders, and reading difficulties.

In addition to their regular academic activities, the rope-skipping group received 10 weeks of fancy rope-skipping training as an after-school program, whereas the control group did not participate in a similar exercise training program.

### 2.2 Study design and procedure

This quasi-experimental study was conducted at Changbin Primary School, Haikou, Hainan Province, China, between October 2022 and December 2022.

#### 2.2.1 Procedure

**Figure 1:**
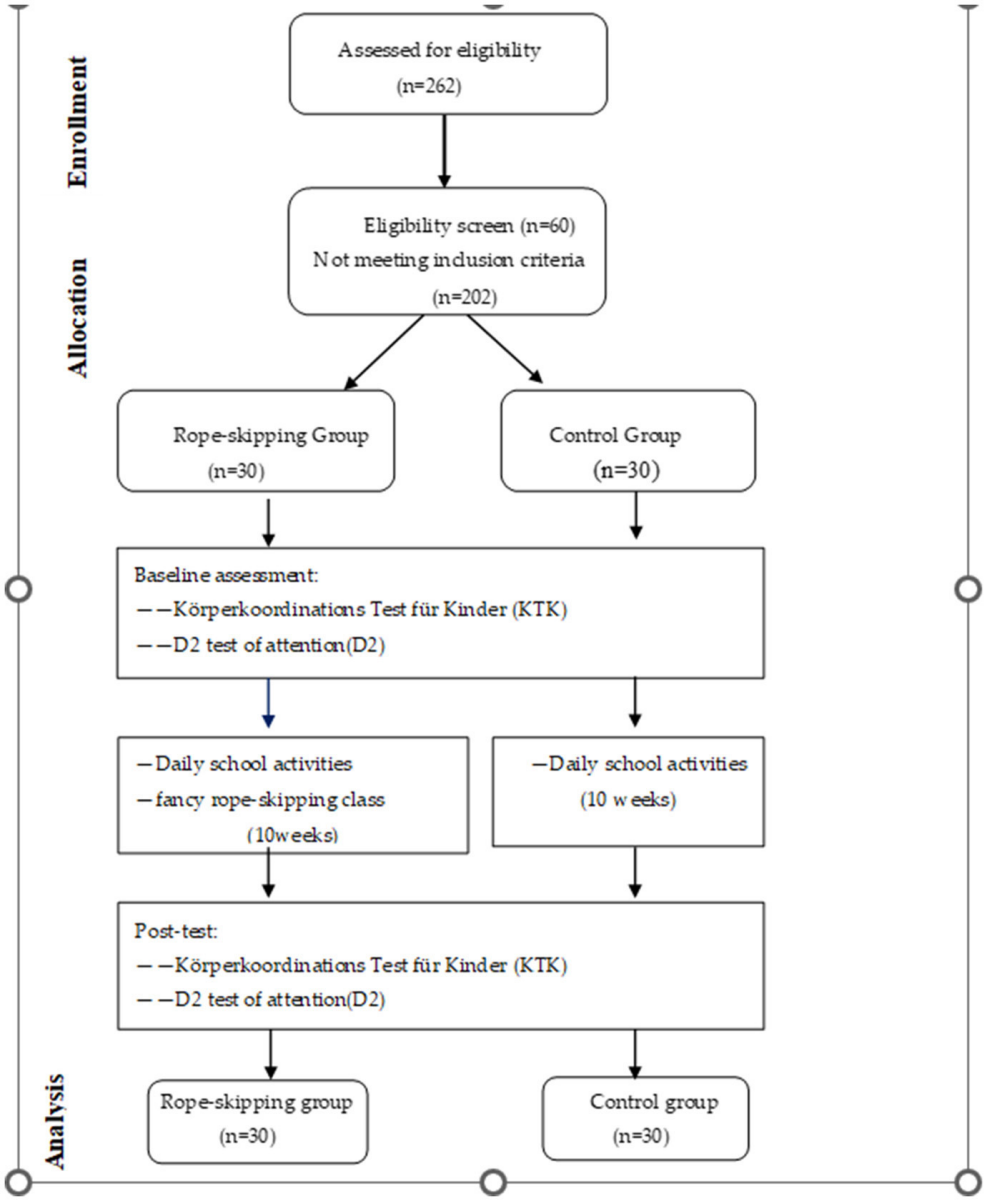
Study procedure.

Under the condition that the basic daily curriculum of the fancy rope-skipping group aligns with that of the control group (comprising 32 cultural classes and three physical education classes per week), the fancy rope-skipping group participates in after-school sessions delivered by professional coaches from the Fancy Rope-Skipping Association on Monday and Wednesday afternoons from 5:00 to 6:00 p.m. These sessions are led by a head professional coach and supported by two teaching assistants, all of whom have undergone standardized training in fancy rope-skipping courses organized by the State Sports Administration. They have obtained certification as fancy rope-skipping coaches and have accumulated over 2 years of experience in teaching fancy rope-skipping. The control group maintained regular study routines without intervention. Motor coordination and selective attention were measured before and after the intervention (Figure 1). The tests were conducted by graduate students who had received specialized training, and all tests were completed within 1 week.

#### 2.2.2 Intervention

The rope-skipping program content was based on Level 1 exercises developed by the Chinese sports agency (Social Sports Guidance Center, 2021) and involved eight instructional exercises: left-right rope swing, double-footed single swing jump, double-footed bicycle jump, jumping jack rope skipping, bow step rope skipping, double-footed left-right rope skipping, basic crossover rope skipping, foot hook ground tap rope skipping, and knee lift rope skipping (Table 1). From the setting of eight teaching contents to the teaching process, we adhere to the principles of progression and differentiated instruction. For instance, in the first lesson, we introduced the basic classroom requirements, the background knowledge of the fancy rope-skipping jump, the equipment of the jump rope, the proper rope length adjustment, and the grasping skills, then focus on learning to use wrist rotation to swing the rope from side to side and practice this coordination with music. In the second lesson, we focus on learning double-footed rope skipping. The instructional process unfolds as follows: firstly, a review of the left-right rope swing from the previous lesson; subsequently, instruction on basic stance, takeoff and landing movements, and stationary double-footed jumps (without a rope) until achieving 10–50 synchronized jumps with music. Next, participants engage in 100 jumps using a cordless jump rope before transitioning to rope skipping, practicing repetitively until proficiency is achieved. We emphasize the coordination of upper body rope swinging with lower body jumping to achieve rhythmic synchronization. Individualized goals are established based on student progress, with the foundational target of completing consecutive double-footed jumps 10 times or more. Each session lasted 60 min and consisted of a 15-min warm-up phase, followed by 35 min of continuous moderate-to-high-intensity physical activity (including the study and practice of rope skipping movements, as well as 5–10 min of basic physical fitness exercises). The session concluded with 10 min of cool-down and stretching exercises. In each class, the learning of rope skipping movements involved explanations of key points such as body posture, jumping timing, wrist movement, hand-foot coordination, breath control, and rhythm. Intensity was monitored by the coach through the Borg rating of perceived exertion (13–14, somewhat hard) (Borg, 1982; Yunting et al., 2017).

**Table 1:** The main content of the fancy jump rope course.

**Weekly**	**Sessions**	**Teaching Content**	**Teaching process and requirements**
1st	1	Left-right rope swing: swing the rope from behind to the front with both arms, swinging the rope to the right side of the body without passing it over the feet; then swing the rope to the left side of the body, with a one-beat-one-movement rhythm. Repeat this sequence on both sides four times to complete the left-right rope swing exercise	(Five sets of 8–10 rep): practice 8–10 perfect rep, stop, rest 5 s and reset. Five rounds, and a 15-s rest between round.
	2	Double-footed single swing jump: hold the rope handle with both hands and swing the rope forward, keeping both feet parallel with a slight gap, jump over the rope, and the rope should make one full circle around the body. Complete the double-footed single swing jump exercise with a one-swing-one-jump rhythm	(Five sets of 5–10 rep): practice 5–10 perfect rep, stop, rest 5 s and reset. Five rounds, and a 15-s rest between round.
2nd	3	Review the previous content	Reviewing left and right rope swinging and jumping rope with both feet. Phase 1, (two sets of 10–15 rep): practice each movement, respectively, 10–15 perfect rep, stop, rest 5 s and reset. Two rounds, and a 15-s rest between round. Phase 2, (two sets of 10–15 rep): connected two movement, 10–15 perfect rep, stop, rest 5 s and reset. Two rounds, and a 20-s rest between round.
	4	Double-footed bicycle jump: start in a stationary double-footed standing position, hold the rope handle with both hands, swing the rope forward once, and then lift each foot one after the other to jump over the rope. Children need to continuously alternate jumping over the rope with each foot, maintaining a one-swing-one-jump rhythm. Repeat this sequence on both the left and right sides four times to complete the double-footed bicycle jump exercise	(Five sets of 4–8 rep): practice 4–8 perfect rep, stop, rest 5 s and reset. Five rounds, and a 15-s rest between round.
3rd	5	Review the previous content	Phase 1, (two sets of 8–10 rep): practice each movement, respectively, 10–15 perfect rep, stop, rest 5 s and reset. Two rounds, and a 15-s rest between round. Phase 2, (two sets of 8–10 rep): connected three movements, 10–15 perfect rep, stop, rest 5 s and reset. Two rounds, and a 15-s rest between round.
	6	Jumping jack rope skipping: start in a static, double-footed standing position, hold the rope handle with both hands, and swing it forward. When both feet are in the air while clearing the rope, quickly spread them apart as they descend, with knees slightly bent to cushion the landing. When the rope makes its second quick contact with the ground, bring both feet together in the air while clearing the rope. Maintain a one-beat-one-movement rhythm to complete the Jumping Jack rope skipping exercise	(Five sets of 4–8 rep): practice 4–8 perfect rep, stop, rest 5 s and reset. Five rounds, and a 15-s rest between round.
4th	7	Review the previous content	Phase 1, (two sets of 8–10 rep): practice each movement, respectively, 10–15 perfect rep, stop, rest 5 s and reset. Two rounds, and a 15-s rest between round. Phase 2, (two sets of 8–10rep): connected four movements, 10–15 perfect rep, stop, rest 5 s and reset. Two rounds, and a 15-s rest between round.
	8	Bow step rope skipping: start in a static, double-footed standing position, hold the rope handle with both hands, and swing it forward. When both feet are in the air while clearing the rope, quickly spread them apart into a front-back bow step motion as they descend. When the rope makes its second quick contact with the ground, bring both feet back together to jump over the rope. Maintain a one-beat-one-movement rhythm, and repeat the bow step motion four times on both sides to complete the Bow Step rope skipping exercise.	(Five sets of 4–8 rep): practice 4–8 perfect rep, stop, rest 5 s and reset. Five rounds, and a 15-s rest between round.
5th	9	Review the previous content	Phase 1, (two sets of 8–10 rep): practice each movement, respectively, 10–15 perfect rep, stop, rest 5 s and reset. Two rounds, and a 15-s rest between round. Phase 2, (two sets of 8–10 rep): connected five movements, 10–15 perfect rep, stop, rest 5 s and reset. Two rounds, and a 15-s rest between round.
	10	Double-footed left-right jump: start in a static, double-footed standing position, hold the rope handle with both hands, and swing it forward. When both feet are in the air while clearing the rope, quickly bring them together and land to the right. On the next rope clearance, jump to the left. Maintain a one-beat-one-movement rhythm, and repeat this sequence four times on both the left and right sides to complete the Double-Footed Left-Right Jump exercise	(Five sets of 4–8 rep): practice 4–8 perfect rep, stop, rest 5 s and reset. Five rounds, and a 15-s rest between round.
6th	11	Review the previous content	Phase 1, (two sets of 8–10 rep): practice each movement, respectively, 10–15 perfect rep, stop, rest 5 s and reset. Two rounds, and a 20-s rest between round. Phase 2, (two sets of 8–10 rep): connected six movements, 10–15 perfect rep, stop, rest 10 s and reset. Two rounds, and a 25 s rest between round.
	12	Basic Cross-Jumping: start with a static and stand on your feet, hold the rope handle with both hands and shake the rope, this action is divided into two beats to complete, the first beat of both hands for the straight shake the rope, the second beat needs to be changed to cross the rope for the change of the two hands, the rhythm of a beat and a move, the two beats were repeated four times, to complete the basic cross-jumping exercises.	(Five sets of 4–8 rep): practice 4–8 perfect rep, stop, rest 5 s and reset. Five rounds, and a 15-s rest between round.
7th	13	Review the previous content	Phase 1, (two sets of 8–10 rep): practice each movement, respectively, 10–15 perfect rep, stop, rest 5 s and reset. Two rounds, and a 20-s rest between round. Phase 2, (two sets of 8–10 rep): connected seven movements, 10–15 perfect rep, stop, rest 10 s and reset. Two rounds, and a 25 s rest between round.
	14	Foot hook ground tap jump: start in a static, double-footed standing position, hold the rope handle with both hands, and swing the rope. In this exercise, while one foot hooks and taps the ground forward, the other foot jumps straight over the rope. Then, on the next rope clearance, alternate and perform the same action. Maintain a one-beat-one-movement rhythm, and repeat this sequence four times with both the left and right feet to complete the Foot Hook Ground Tap Jump exercise.	(Five sets of 4–8 rep): Practice 4–8 perfect rep, stop, rest 5 s and reset. Five rounds, and a 15-s rest between round.
8th	15	Review the previous content	Phase 1, (two sets of 8–10 rep): practice each movement, respectively, 10–15 perfect rep, stop, rest 5 s and reset. Two rounds, and a 20-s rest between round. Phase 2, (two sets of 8–10 rep): connected eight movements, 10–15 perfect rep, stop, rest 10 s and reset. Two rounds, and a 25 s rest between round.
	16	Knee lift jump: start in a static, double-footed standing position, hold the rope handle with both hands, and swing the rope. When both feet are in the air while clearing the rope, lift one knee forward while the other foot jumps straight over the rope. On the next rope clearance, alternate and perform the same action. Maintain a one-beat-one-movement rhythm, and repeat this sequence four times with both the left and right feet to complete the Knee Lift Jump exercise.	(Five sets of 4–8 rep): practice 4–8 perfect rep, stop, rest 5 s and reset. Five rounds, and a 15-s rest between round.
9th	17	Review the previous content	Phase 1, (two sets of 8–10 rep): practice each movement, respectively, 10–15 perfect rep, stop, rest 5 s and reset. Two rounds, and a 20-s rest between round. Phase 2, (two sets of 8–10 rep): connected night movements, 10–15 perfect rep, stop, rest 10 s and reset. Two rounds, and a 25 s rest between round.
	18	Intensive and sequential practice of the first five exercises	Phase 1, (two sets of 8–10 rep): practice previous four movements, respectively, 10–15 perfect rep, stop, rest 5 s and reset. Two rounds, and a 20-s rest between round. Phase 2, (two sets of 8–10 rep): connected night movements, 10–15 perfect rep, stop, rest 10 s and reset. Two rounds, and a 25 s rest between round.
10th	19	Intensive and sequential practice of the last four exercises	Phase 1, (two sets of 8–10 rep): Practice last five movements, respectively, 10–15 perfect rep, stop, rest 5 s and reset. Two rounds, and a 20-s rest between round. Phase 2, (two sets of 8–10 rep): connected night movements, 10–15 perfect rep, stop, rest 10 s and reset. Two rounds, and a 25 s rest between round.
	20	To demonstrate a simple creative rope skipping routine	Five-min preparation time, draw lots by group to show the pattern rope skipping simple creation routine, 30 s−1 min.

#### 2.2.3 Outcome measures

- Motor Coordination Measurement. The KTK was developed in Germany in 1974. It is a standardized assessment tool designed to evaluate and measure the motor coordination skills of children aged 5–14 years old (Kiphard and Schilling, 1974). It has been used and showed good reliability and validity worldwide (Vandorpe et al., 2011; Moreira et al., 2019), including in studies on Chinese children (Song, 2020). The KTK demonstrates a good test-retest reliability (0.76–0.85), which is similar to Li et al.'s (2023) study. The KTK assessment was conducted in this study and its scoring adhered to the guidelines provided in the manual (Kiphard and Schilling, 2007), which are outlined below.

(1) Walking backwards: Participants were instructed to walk backward on three different wooden planks, which were 5 cm above the ground and 3 m in length, with widths of 6, 4.5, and 3 cm, respectively. Scoring was determined based on the total number of backward steps.
(2) Hopping for height: Using 60 × 20 × 5 cm foam mats as obstacles, participants were directed to execute one-legged jumps with the objective of clearing the foam mats while landing on the same foot. Scoring was determined based on the number of successfully cleared foam mats and the total number of attempts.
(3) Jumping sideways: On a 100 × 60 cm rectangular wooden board divided into two equal sections by a 60 × 4 × 2 cm obstacle placed in the center, participants performed lateral two-legged jumps from side to side. The objective was to tally the number of jumps completed in a 15-s window, with two attempts granted. The final score was derived by adding the scores of both attempts.
(4) Moving sideways: Participants were instructed to stand on one side of a divided wooden board with an obstacle, and their task was to perform sideways movements by lifting one foot at a time and placing it on the opposite side of the obstacle. The test measures the number of lateral movements completed in a 20-s timeframe, with two attempts. This score was added twice. Finally, the overall results were added to the four sub-item results.

- Selective attention measurement. The d2 Test of Attention is a widely used psychological assessment tool designed to measure selective attention and concentration (Brickenkamp and Zillmer, 1998). The scale exhibits high reliability and validity, it showed good test-rest reliability (0.80–0.91), as similar to previous research (Lee et al., 2018). The d2 Test of Attention consists of a grid containing 14 rows and 47 columns housing 658 characters. These characters are composed of four types: “d,” “b,” “p,” and “q.” Each of these characters is accompanied by zero, one, or two short vertical dashes or dots above, below, or above and below them (′, ″). During the test, participants were instructed to find and mark, within a maximum of 20 s for each row, the characters in each row that had two vertical dashes or dots above them, while ignoring all other characters.

The scale has five dimensions calculated based on the foundation parameters of the total number of items (TN), *attention concentration (TN-E)*, concentration performance (CP), *the error percentage (E%), and the fluctuation rate (FR)*. Regarding dimensions, the first is TN, which denotes the total number of characters processed within a specified time, including correct responses, omissions, and errors. The second is a TN-E, which is the total number of symbols per row minus the total number of errors [E = E1 + E2, errors of omission (E1), errors of commission (E2)]. The third is CP, which is the total number of correct responses minus E2. The fourth is the E%, which is the percentages of E1 and E2 within the TN in a row. Finally, the FR refers to the variance between the highest and lowest counts of tagged characters.

### 2.3 Statistical analysis

Data were analyzed using SPSS Statistics version 26.0 (IBM). The Shapiro–Wilk test was used to assess the normal distribution, and the diverse evaluation parameters were represented as mean ± standard deviation (M + SD). A 2 (rope-skipping and control groups) × 2 (baseline and after 10 weeks) mixed-model ANOVA was used to evaluate the impact of the intervention on the KTK and d2 Test of Attention scores. The simple main effects of time were analyzed to demonstrate the mean change in scores over the 10 weeks for each group. The changes observed across these 10 weeks were subsequently presented as a percentage change (% Δ). The effect size for the interaction effect was assessed using partial eta square (ηp^2^), with values categorized as small (0.01), medium (0.06), and large (0.14) (Lakens, 2013). Statistical significance was set at a *p* < 0.05.

## 3 Results

Participants' demographic characteristics are shown in Table 2.

**Table 2:** Demographic characteristics of the two groups.

	**Rope-skipping group**	**Control group**
Age (Mean ± SD)	8.07 ± 0.69	8.03 ± 0.85
**Sex**		
Male *n*, (%)	10 (33.3%)	11 (36.7%)
Female *n*, (%)	20 (66.7%)	19 (63.3%)
Height	127.23 ± 4.86	127.50 ± 6.56
Weight	25.30 ± 6.56	24.00 ± 3.82
BMI	15.52 ± 2.10	14.69 ± 1.52

### 3.1 Comparison of KTK scores after interventions

The results showed that KTK total scores were significantly higher in the rope-skipping group (% Δ = 8.29, *p* < 0.001) than in the control group (% Δ =1.89, *p* < 0.001) after the 10-week intervention. Additionally, there was a significant interaction effect between time and the intervention [*F*_(1, 58)_ = 7.931, *p* = 0.007, ηp^2^ = 0.12, medium]. Similar changes were observed in the hopping for height, jumping sideways, and moving sideways. A comparison of the scores for the KTK individual items between groups is presented in Table 3.

**Table 3:** The KTK scores at pre- and post-test for the rope-skipping and control groups.

**Outcome**	**Pre-test**	**Post-test**	**% Δ**	**A group-by-time interaction effect**		
**Group**	**Mean ±SD**	**Mean ±SD**		***F*_(1, 58)_**	***p***	**Partial η^2^**
**Walking backwards**						
RSG	35.97 ± 12.21	35.50 ± 9.63	−1.31	0.161	0.69	0.003 (very small)
CON	40.20 ± 8.96	40.70 ±8.21	**-**1.24			
**Hopping for height**						
RSG	42.90± 12.86	48.73 ± 10.89	13.59^**^	5.79	0.019^*^	0.091 (medium)
CON	42.87 ±10.67	44.53 ± 6.85	3.87			
**Jumping sideways**						
RSG	45.03 ± 5.62	49.93± 5.63	10.88^**^	11.809	0.001^*^	0.169 (large)
CON	43.37 ± 5.04	43.77 ± 4.70	0.92			
**Moving sideways**						
RSG	15.23 ± 2.13	16.50 ± 1.55	8.33^*^	5.678	0.02^*^	0.089 (medium)
CON	14.80 ± 2.04	14.90 ± 1.42	0.68			
**Total score**						
RSG	139.13 ± 26.81	150.67 ± 20.59	8.29^**^	7.931	0.007^*^	0.120 (medium)
CON	141.23± 16.31	143.90 ± 12.62	1.89^**^			

RSG, rope-skipping group; CON, control group.

^*^p < 0.05, ^**^p < 0.01.

### 3.2 Comparison of d2 scores after interventions

When comparing the changes in d2 scores between the two groups after the 10-week intervention, we found that E% and FR decreased significantly in the rope-skipping group, while the control group showed no significant changes. The CP score improved significantly in the rope-skipping group; however, the changes in the control group were not significant. However, the difference between the two groups was only significant in the TN-E category, and we observed a significant interaction effect between time and the intervention [*F*_(1, 58)_ = 4.421, *p* = 0.004, ηp^2^ = 0.071, medium]. A comparison of the scores by group for the d2 Test of Attention individual items is presented in Table 4.

**Table 4:** The d2 test scores at pre- and post-test for the rope-skipping and control groups.

**Outcome**	**Pre-test**	**Post-test**	**% Δ**	**A group-by-time interaction effect**		
**Group**	**Mean ±SD**	**Mean ±SD**		***F*_(1, 58)_**	***p***	**Partial η^2^**
**TN**						
RSG	623.53 ± 61.63	650.17± 21.68	4.33	3.465	0.068	0.056 (small)
CON	636.07 ± 42.28	632.37 ± 37.52	−0.63			
**TN-E**						
RSG	580.90 ± 64.28	614.80 ± 31.84	5.86	4.421	0.040^*^	0.071 (medium)
CON	593.80 ± 46.44	591.77 ± 42.98	−0.34			
**E%**						
RSG	6.90 ± 3.25	5.47± 2.96	−20.72^**^	3.014	0.088	0.049 (small)
CON	6.68 ± 2.28	6.48 ± 2.21	−2.99			
**CP**						
RSG	53.37 ± 20.34	60.63 ± 18.60	13.60^**^	1.778	0.188	0.030 (small)
CON	53.73 ± 13.69	55.40 ± 12.81	3.12			
**FR**						
RSG	6.17 ± 2.17	4.93 ± 1.72	−20.09^*^	2.870	0.096	0.047 (small)
CON	5.83 ± 1.80	5.57± 1.41	−4.50			

RSG, rope-skipping group; CON, control group.

^*^p < 0.05, ^**^p < 0.01.

## 4 Discussion

This study evaluated the effectiveness of a 10-week fancy rope-skipping intervention on motor coordination and selective attention in 7–9-year-old children. Compared to the control group, the rope-skipping group showed significant improvements in overall motor coordination scores, as well as in specific aspects such as hopping for height, jumping sideways, and moving sideways. Regarding the d2 test measures, in the control group, no significant improvements were observed after the intervention, whereas the rope-skipping group demonstrated improvements in E%, CP, and FR compared to the baseline. Still, only the TN-E category showed a change in the rope-skipping group that was significantly higher than that of the control group. These findings indicate that our 10-week fancy rope-skipping intervention may potentially enhance the development of motor coordination in children aged 7–9 years. Furthermore, the intervention seems to influence attention accuracy; however, its effects on other aspects of selective attention remain uncertain.

Motor coordination involves a series of brain processes, including sensory input, perceptual and cognitive processing, and motor production, all of which work together to achieve precise and coordinated movements (Iorga et al., 2023). Fancy rope-skipping typically involves a variety of complex rope-skipping movements that incorporate changes in posture and jumps, such as single swings, double swings, leg hooks, knee raises, and other transitions. That is, it is a physical activity that requires highly coordinated movements of the upper and lower body muscles to regain balance and propulsion, while synchronizing with the rhythm of the music and the rope swing. In this study, improvements in motor coordination, such as hopping for height, were assessed in relation to the coordination of the lower limbs and to explosive power. From a muscle contraction perspective, rope jumping is considered a stretch-shortening cycle exercise, which is a key physiological process for generating explosive power and rapid movements (Miyaguchi et al., 2014). During a stretch-shortening cycle, muscles are stretched and quickly shortened, releasing stored elastic energy to produce a significant force. This process involves neural control and coordination to ensure that muscle fibers shorten at the appropriate time for the desired movement and force production (Aeles and Vanwanseele, 2019). Chen and Wu (2022) showed that 8 weeks of rope-skipping training can improve lower limb coordination and strength, and enhancing standing long jump performance in male college students. A systematic review found that compared to regular physical education classes, jump rope exercises resulted in better improvement of coordination in preadolescent girls (Zhao et al., 2023).

Regarding the procedures in the KTK assessment, the jumping sideways procedure reflects coordination and explosiveness during lateral jumps, while the moving sideways procedure assesses coordination and flexibility during lateral movements. The observed improvements in these measures suggest an association between the 10-week fancy rope-skipping training and improvements in children's coordination and stretch-shortening cycle capabilities. These findings align with those in a past study, wherein 8 weeks of rope-skipping exercises in soccer training enhanced overall motor coordination in preadolescent soccer players (Trecroci et al., 2015). However, the improvement in backward walking was not significant, possibly because the primary focus of the fancy rope-skipping content in this study was on forward and lateral jumping movements, which emphasize forward and lateral balance and coordination. Conversely, backward walking places a stronger emphasis on reverse movement control.

Prior research has suggested that complex exercise intervention programs can be utilized to stimulate both the physical and higher-order cognitive skills of pre-adolescent children (Greco et al., 2023). Fancy rope-skipping involves a wide range of movements and techniques, ranging from basic single jumps to more complex maneuvers such as crossovers and rotations. These movements require flexibility in switching and adjusting between them, as well as active cognitive engagement. The d2 Test of Attention assesses an individual's ability to concentrate attention and make rapid and accurate target identifications and markings when faced with distracting information (Gutiérrez-Hernández et al., 2021). Thus, it reflects visual perception speed and concentration abilities and is not related to intelligence. In this study, the rope-skipping group exhibited a significant decrease in E% and FR after the intervention. A lower E% generally indicates higher attentional accuracy, while a lower FR suggests that an individual can maintain their focus more consistently. The significant improvement in the CP index in the rope-skipping group indicates an enhanced ability to maintain focus within a limited timeframe. Meanwhile, in the control group, the changes in these indicators were not significant. When comparing groups, the significant increase in TE-E reflected better attention performance in the children concerning target identification and marking. A similar study by Buchele Harris et al. (2018) examined the effects of daily 6-min coordinated-bilateral physical activity breaks over a 4-week period in fifth-grade students and found that the intervention significantly increased TN, TN-E, CP, and FR.

However, there has been relatively limited research on the effects of exercise interventions on selective attention, and most related studies have focused on the immediate effects of these interventions. Research by Ligeza et al. (2023), demonstrated that a single session of vigorous-intensity exercise improved neural processing related to selective attention. Meanwhile, Altermann and Gröpel (2023) found that moderate-to-high-intensity endurance, strength, and coordination exercises lasting 25 min each led to similar improvements in selective attention, and that coordination exercises did not show a distinct advantage. It was speculated that these similar improvements across exercises may be due to the longer duration and greater intensity of these exercises, which generally elevate brain activity levels (including prefrontal cortex structures) and make changes evoked by the exercise no longer closely tied to the complexity of the exercise, but rather related to increased brain oxygenation and activation. Guillamón et al. (2021) found that engaging in short-term aerobic exercise may have a positive acute effect on selective attention. Hernández et al. (2021) suggested that aerobic fitness is beneficial for selective attention and inhibition, particularly during childhood.

Based on these findings, further researched have also investigated the underlying mechanisms through which aerobic exercise impacts selective attention. Dodwell et al. (2021) investigated the effects of different intensities of aerobic exercise on attention control mechanisms using event-related potentials (ERPs). The study found that moderate-intensity aerobic exercise can eliminate interference effects in attention control mechanisms and elicit a potential related to distractors, moderate exercise levels are optimal for enhancing cognitive processing efficiency, particularly in attentional allocation. Biochemically, it is found that engaging in rhythmic long-rope jumping enhances cognitive performance, particularly attention, by activating the central norepinephrinergic system, Norepinephrine plays a role in regulating both the dorsal and ventral attention networks, influencing the reorienting and shifting of attention (Yamashita and Yamamoto, 2021).

Generally, our results partially corroborate past evidence, which have suggested that coordination exercises may have a significant impact on children's concentration and performance accuracy (Gallotta et al., 2015). Specifically, the positive influence we observed of the rope-skipping training on attention performance in children aged 7–9 years may be related to the activation of specific shared neural structures in cognitive and motor coordination, such as the cerebellum and prefrontal cortex (Henschke and Pakanm, 2023). Based on these assertions, this study's findings concur with past evidence suggesting that incorporating coordination exercises into the lives of children and adolescents can enhance their utilization of attentional resources and optimize the efficiency of their neural cognitive processing (Shi and Feng, 2022). Therefore, in the physical education of school-aged children, the use of tasks and exercises that stimulate cognitive engagement may provide effective pathways for enhancing cognitive function.

This study has several limitations. Firstly, the d2 analysis did not control for potential confounding factors such as age, educational background, and additional neuropsychological assessments, which could influence the results. Future studies should include these controls to enhance the robustness of the findings. Secondly, the use of a quasi-experimental design without randomization may have introduced potential biases and limited the ability to draw causal conclusions. Future research should aim to employ a randomized controlled trial design to minimize these biases and strengthen the validity of the results. Additionally, the study's reliance on psychometric assessments may have inherent limitations in accuracy and objectivity. Incorporating more objective measures, such as neuroimaging assessments, could provide a more comprehensive understanding of the effects of fancy rope-skipping on motor coordination and selective attention. Despite these limitations, the findings provide valuable insights into the potential benefits of fancy rope-skipping for children's cognitive and coordination development. Future research should continue to explore these relationships with more rigorous methodologies and larger sample sizes to confirm and extend these findings.

## 5 Conclusions

In conclusion, our study suggested that a 10-week fancy rope-skipping intervention may potentially enhance motor coordination and selective attention accuracy in children aged 7–9 years. The fancy rope-skipping intervention offers a simple, cost-effective, and engaging way to promote physical activity while simultaneously enhancing cognitive functions such as attention and coordination. Incorporating such interventions into school curricula or extracurricular activities could contribute to the holistic development of children, fostering not only physical fitness but also cognitive skills essential for academic success and overall wellbeing. Furthermore, our study underscores the importance of regular physical activity programs tailored to children's developmental needs. By integrating activities that target both motor coordination and selective attention, educators and practitioners can support the comprehensive development of children's physical and cognitive abilities.

## Data availability statement

The data are available from the corresponding author on reasonable request.

## Ethics statement

The studies involving humans were approved by Ethics Committee of Hainan Institute of Sports Science (GT-QM-03). The studies were conducted in accordance with the local legislation and institutional requirements. Written informed consent for participation in this study was provided by the participants' legal guardians/next of kin.

## Author contributions

LD: Methodology, Writing – original draft. HW: Conceptualization, Writing – original draft, Writing – review & editing. HR: Supervision, Writing – review & editing. DX: Investigation, Writing – review & editing. SP: Formal analysis, Writing – review & editing. MS: Data curation, Writing – review & editing.
